# Machine learning reveals two heterogeneous subtypes to assist immune therapy based on lipid metabolism in lung adenocarcinoma

**DOI:** 10.3389/fimmu.2022.1022149

**Published:** 2022-09-27

**Authors:** Xuyu Gu, Shiyou Wei, Zhixin Li, Huan Xu

**Affiliations:** ^1^ School of Medicine, Southeast University, Nanjing, China; ^2^ Department of Anesthesiology, Shanghai Pulmonary Hospital, School of Medicine, Tongji University, Shanghai, China; ^3^ Department of Thoracic Surgery, Shanghai Pulmonary Hospital, School of Medicine, Tongji University, Shanghai, China

**Keywords:** lung adenocarcinoma, machine learning, tumor microenvironment, lipid metabolism, immunotherapy

## Abstract

**Background:**

Lipid metabolism pivotally contributes to the incidence and development of lung adenocarcinoma (LUAD). The interaction of lipid metabolism and tumor microenvironment (TME) has become a new research direction.

**Methods:**

Using the 1107 LUAD records from the Cancer Genome Atlas (TCGA) and Gene Expression Omnibus (GEO) databases, a comprehensive exploration was performed on the heterogeneous lipid metabolism subtypes based on lipid metabolism genes (LMGs) and immune-related genes (LRGs). The clinical significance, functional status, TME interaction and genomic changes of different subtypes were further studied. A new scoring system, lipid-immune score (LIS), was developed and validated.

**Results:**

Two heterogeneous subtypes, which express more LMGs and show the characteristics of tumor metabolism and proliferation, are defined as lipid metabolism phenotypes. The prognosis of lipid metabolism phenotype is poor, and it is more common in patients with tumor progression. Expressing more IRGs, enrichment of immunoactive pathways and infiltration of effector immune cells are defined as immunoactive phenotypes. The immunoactive phenotype has a better prognosis and stronger anti-tumor immunity and is more sensitive to immunotherapy. In addition, KEAP1 is a driving mutant gene in the lipid metabolism subtype. Finally, LIS was developed and confirmed to be a robust predictor of overall survival (OS) and immunotherapy in LUAD patients.

**Conclusion:**

Two heterogeneous subtypes of LUAD (lipid metabolism subtype and immune activity subtype) were identified to evaluate prognosis and immunotherapy sensitivity. Our research promotes the understanding of the interaction between lipid metabolism and TME and offers a novel direction for clinical management and precision therapy aimed to LUAD patients.

## Introduction

As the most frequent malignancy, lung cancer causes the highest cancer-related deaths around the world (1). Lung cancer can appear in different histological types among which, non-small cell lung cancer (NSCLC) is the most common type with about 85% proportion of all lung cancer patients (2). Lung adenocarcinoma (LUAD) is the most abundant subtype of NSCLC, accounting for about 55% (3). LUAD is a heterogeneous disease with different clinical prognosis and drug response results. It is worth noting that despite the great progress in clinical diagnostic methods and multimodal treating approaches, the 5-year overall survival (OS) rate of patients with advanced lung cancer has remained very low (4). Therefore, LUAD patients are still in an urgent need for new early diagnosis and clinical intervention methods.

During cancer occurrence and progression, the immune system and the tumor cells are in complex interaction. On one hand, demand for local nutrition and oxygen highly increases due to fast proliferation of tumor cells. On the other hand, the same reason causes poor local vascularization, resulting in acidosis and hypoxia in the tumor microenvironment (TME) as well as local glucose deficient (5–7). Eventually, lipids existing in the TME begin to be used main as the alternative source of energy in both tumor tissues and immune cells to compensate for the energy shortages (8). Lipids also contribute to the biofilm formation, supplying the biomass production, and mediating some complex signaling pathways contributing to the growth and migration of cancer cells (9). In addition, the affinity of cancerous cells for lipids and cholesterol increases, directly leading to lipid accumulation in the TME and developing malignancy in the tumor tissues (10). Although, the lipid metabolic reprogramming and dysfunction as well as its dual impact in the TME and immune responses to tumor is not exactly recognized yet. Such further elaboration is essentially required for developing specific treatments based on the anti-tumor immune responses.

The present study aimed to survey the crosstalk between lipid metabolism and tumor immune response in LUAD patients and identified two heterogeneous subtypes (lipid metabolism subtype and immune activity subtype). These two subtypes show specific differences in clinical outcomes, biological functions, immune infiltration and genomic variation. In addition, a lipid-immune score (LIS) was developed and validated, which shows significant advantages in predicting prognosis and immunotherapy response. In conclusion, our work strengthens the understanding of the complex role between lipid metabolism and immune system in LUAD and provides a new perspective and reference for the accurate prediction and immunotherapy of LUAD patients.

## Methods

### Data extraction

Transcriptome RNA-seq data (HT-seq FPKM), mutation data (mutect2 tool), copy number variation (CNV), and their corresponding clinical information (from Cancer Genome Atlas-lung adenocarcinoma, tcga-LUAD queue) were obtained (https://portal.gdc.cancer.gov/repository). After excluding patients who lost follow-up and clinical information, 492 LUAD samples were collected. These data were used as discovery queues after Transcripts per million (TPM) standardization. In addition, three independent data sets from the GEO database were collected, including GSE30219 from GPL570 platform, GSE42127 from GPL6884 platform, and GSE72094 from GPL15048 platform. In order to prevent the batch effect of chips, three GEO data sets were combined through the combat function of the “sva” package and the data were log2 standardized (11). Finally, a total of 615 GEO meta queues containing LUAD samples with complete clinical information were used as external validation queues. Finally, two immunotherapy cohorts were collected to verify the model’s prognostic power: NSCLC cohort GSE135222 receiving Programmed Death-1(PD-1) treatment, including 27 patients (12) and Imvigor210, a cohort of advanced urothelial carcinoma cases undergoing anti-Programmed Cell Death-Ligand 1 (PD-L1) immunotherapy, including 298 patients (13).

### Identification of lipid and immune subtypes of LUAD

Lipid metabolism genes (LMGs) were obtained from the Molecular Signatures Database (MSigDB) (http://www.gsea-msigdb.org/gsea/index.jsp), containing 1426 LMGs (14). The immune-related genes (IRGs) were obtained from the “ImmPort” database (https://www.immport.org/resources) (15). It contains a total of 1638 IRGs defined as functional and immune related. A detailed list in **Table S1** to indicate the lipid genes and immune genes we used. First, LMGs and IRGs with independent prognostic efficacy were evaluated by univariate Cox regression analysis, and candidate genes were identified according to the threshold of p < 0.05. According to the transcriptional map of candidate genes, consensus clustering was conducted in the discovery queue and validation queue through the ConsensusClusterPlus package (16). Pam unsupervised clustering algorithm was adopted in this analysis, and 1000 iterations were carried out based on Euclidean distance. Eighty per cent of the samples were randomly selected in each iteration. The number of clusters was set to 2-5, and the optimal cluster number was jointly determined using the consensus matrix and cumulative distribution function (CDF).

### Functional enrichment and immune infiltration analysis

Significant differentially expressed genes (DEGs) between subgroups were identified by ‘limma’ package in R program according to the threshold of False Discovery Rate (FDR) < 0.05 and fold change (FC) > 2. The functional enrichment of DEGs was achieved using metascape (www.metascape.org/) database. Gene Set Enrichment Analysis (GSEA) was conducted among subgroups and significantly altered pathways were selected using Kyoto Encyclopedia of Genes and Genomes (KEGG) by p < 0.05.

Based on the previously published molecular markers, ssGSEA analysis was performed using the ‘gsva’ package in R program to evaluate the biological pathway activity of the samples which included angiogenesis, epithelial-mesenchymal transition (EMT), myoid inflammation, and molecular markers of other immune related pathways (17–20). Molecular markers of hypoxia were collected from MSigDB (14). Detailed pathway gene markers were displayed in **Table S2**. The relative infiltration abundance of 22 different immune cell types was evaluated by ‘cibersoft’ package in R program (21). The immune activity and tumor purity of tumor samples were evaluated by Estimate algorithm (22). Finally, the microsatellite instability (MSI) score, indel neoantigens and SNV neoantigens of samples from the study of Thorsson et al. (23).

### Analysis of the genome variation map between subgroups

The mutation data was processed with ‘maftools’ package in R package. First, the total number of mutations in the sample was measured, and then, the genes with the minimum mutation number > 30 were identified. The difference of mutation frequency of high-frequency mutation genes between the two subgroups was compared using the chi square test and visualized with maftools (24). CNV data were processed by Gistic 2.0 software. Based on the threshold of 0.2, significantly amplified and deleted chromosome segments were identified, and CNV differences on chromosome arms were evaluated. The CNV results were visualized by ‘ggplot2’ R package.

### Constructing lipid-immune score

DEGs contained in all cohorts were selected for further analysis based on the above identified DEGs between the two subtypes. Univariate Cox regression analysis revealed the prognostic value of these genes. Subsequently, genes with statistical significance (p < 0.05) were incorporated into the Cox proportional hazard model with Least absolute shrinkage and selection operator (Lasso) penalty, and 300 iterative searches were carried out to find the most robust model. In order to prevent over fitting, five-cross validation was set up. The model with the highest frequency in 300 iterations was used as the final prognostic model and the lipid-immune score (LIS) was generated according to the formula: *LIS* = ∑*iCoefficient*(*mRNA_i_*) × *Expression*(*mRNA_i_*). The ‘survcomp’ package in R program was used to calculate the consistency C index and evaluate the prognostic value of the risk score (RS) in the training and verification sets. The higher C index indicates the more accurate prognostic power of the model (25). The high-risk group and low-risk group were divided based on their median FRS, and the prognostic value of the risk model was calculated using Kaplan-Meier (KM) survival curve, univariate and multivariate Cox regression, time-dependent ROC curve (tROC), and subgroup analysis system.

### Predicting immunotherapy response

According to previous studies, the immunophenoscore (IPS) of the sample was calculated. Briefly, IPS is calculated from transcriptomic data of representative genes for different immunophenotypes and normalizes the final result to 0-10. Samples were positively weighted according to effective immune cells and negatively weighted according to suppressive immune cells, and then applied Z-score averaged. Z-score ≥ 3 was defined as IPS10 and Z-score ≤ 0 was defined as IPS0. The higher the IPS, the better the immunotherapy response (26). The Tumor Immune Dysfunction and Exclusion (TIDE) algorithm (http://tide.dfci.harvard.edu) was applied to predict the patients’ response to the anti-PD-1 and anti-CTLA-4 treatments (27–30). Finally, the predictive power of LIS was evaluated in two external immunotherapy cohorts (GSE135222 and Imvigor210).

### Statistical analysis

Pearson chi square or Fisher exact tests were applied to compare categorical variables. The continuous variables were compared between the two groups by Wilcoxon rank sum test. The KM curve was drawn by ‘survminer’ package and the tROC analysis was carried out by ‘survivalROC’ package both in R program. The univariate and multivariate Cox regression was completed by ‘survival’ package in R program. The ‘rms’ package in R was used to draw nomograms and calibration curves, and decision curve analysis (DCA) was carried out through DCA package (31). The ROC curve used to predict immunotherapy was performed by the ‘pROC’ package. Two tailed p < 0.05 was considered statistically significant unless otherwise specified.

## Results

### Parsing LMGs and IRGs in LUAD

**Figure 1:**
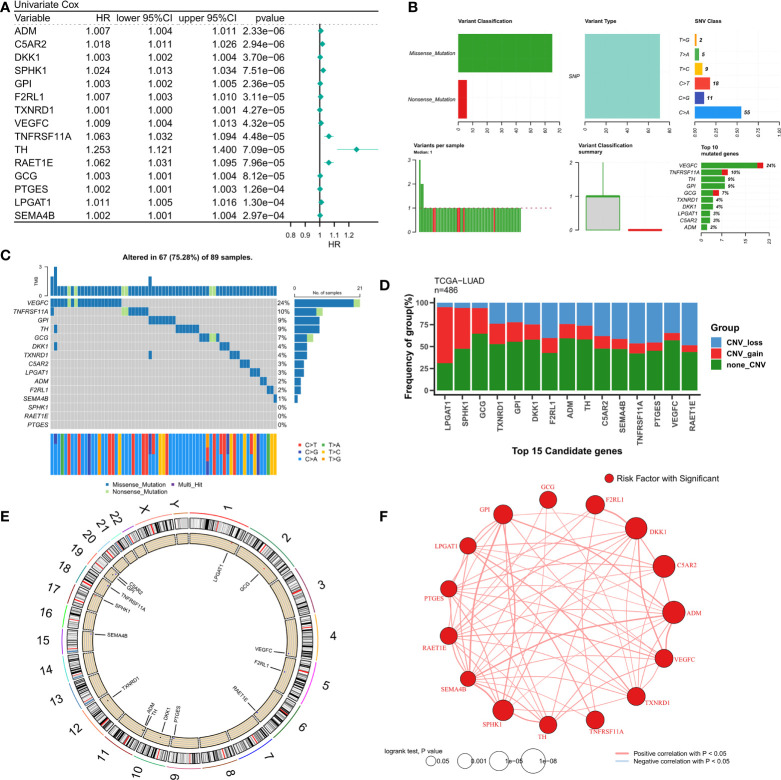
Genomic map of LMGs in LUAD. **(A)** univariate Cox regression analysis revealed the prognostic LMGs of Top15. **(B)** Summary of LMGs mutation events in tcga-LUAD. **(C)** Oncoplot showed the mutation map of LMGs. **(D)** Summary of CNV events of LMGs in tcga-LUAD. **(E)** The circle diagram shows the overall CNV characteristics of LMGs on chromosomes. **(F)** Correlation network of LMGs.

The design of our study is shown in **Figure S1**. Univariate Cox regression analysis displayed 155 LMGs and IRGs with prognostic value (p < 0.05). The forest map showed the prognostic candidate genes of top15 (**Figure 1A**). Detailed Cox results are provided in **Table S3**. **Figure 1B** summarizes the mutation of top15 candidate genes. Specifically, the mutation type is single nucleotide mutation, and the genes with the highest mutation frequency are VEGFC (24%) and tnfrsf11a (10%). The waterfall diagram shows their mutation map in the tcga-LUAD cohort (**Figure 1C**). The histogram summarizes the CNV of the top15 candidate genes in tcga-LUAD, and the results show that they have a wide range of CNV events. Lpgat1 was the most amplified gene, and raet1e was the most deleted gene (**Figure 1D**). The circle chart shows the overall CNV of the top15 candidate gene on the chromosome (**Figure 1E**). Finally, the interaction of top15 candidate genes was analyzed, and the correlation network showed that they were highly positively correlated (**Figure 1F**).

### Identification of lipid and immune subtypes

**Figure 2:**
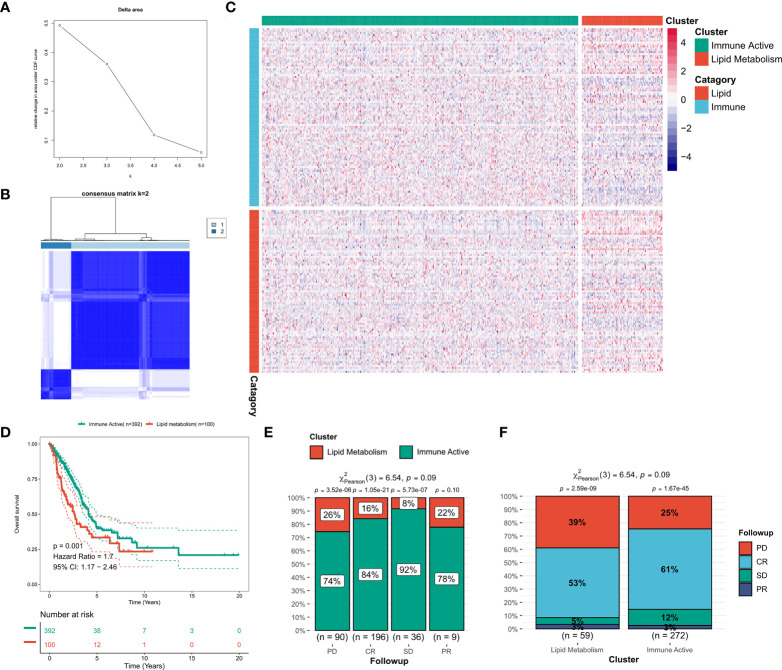
Identification of lipid and immune subtypes. **(A)** CDF curve of consensus matrix of different K **(B)** Consensus matrix when k = 2. **(C)** Expression heat maps of LMGs and IRGs in the two subtypes. **(D)** Survival curve of two subtypes in TCGA cohort. **(E)** The proportion of subtypes in LUAD patients with different outcomes. **(F)** Clinical outcomes of patients with different subtypes of LUAD.

Consensus clustering was performed on the discovery queue and GEO meta queue from tcga-LUAD by ConsensusClusterPlus. According to the CDF curve of consensus score, k = 2 was found to be the best choice (**Figure 2A**, **Figure S2A**). The consensus matrix also confirmed this result (**Figure 2B**, **Figure S2B**). Based on the transcriptional profiles of candidate LMGs and IRGs, lipid metabolism subtypes and immune activity subtypes were defined (**Figure 2C**, **Figure S2C**). IRGs were significantly increased in immunoactive subtypes, while LMGs were significantly increased in lipid metabolism subtypes. According to the survival analysis, lipid metabolism subtypes in the cohort was significantly worse compared to that of immune activity subtypes (p = 0.001, **Figure 2D**). A worse clinical outcome of lipid metabolism subtypes was confirmed in the validation cohort (p < 0.001, **Figure S2D**). In addition, the tcga-LUAD cohort had more detailed clinical follow-up information. There was a significant increase in patients with disease progression in the lipid metabolism subtype (**Figures 2E, F**).

### Biological function difference between two subtypes

**Figure 3:**
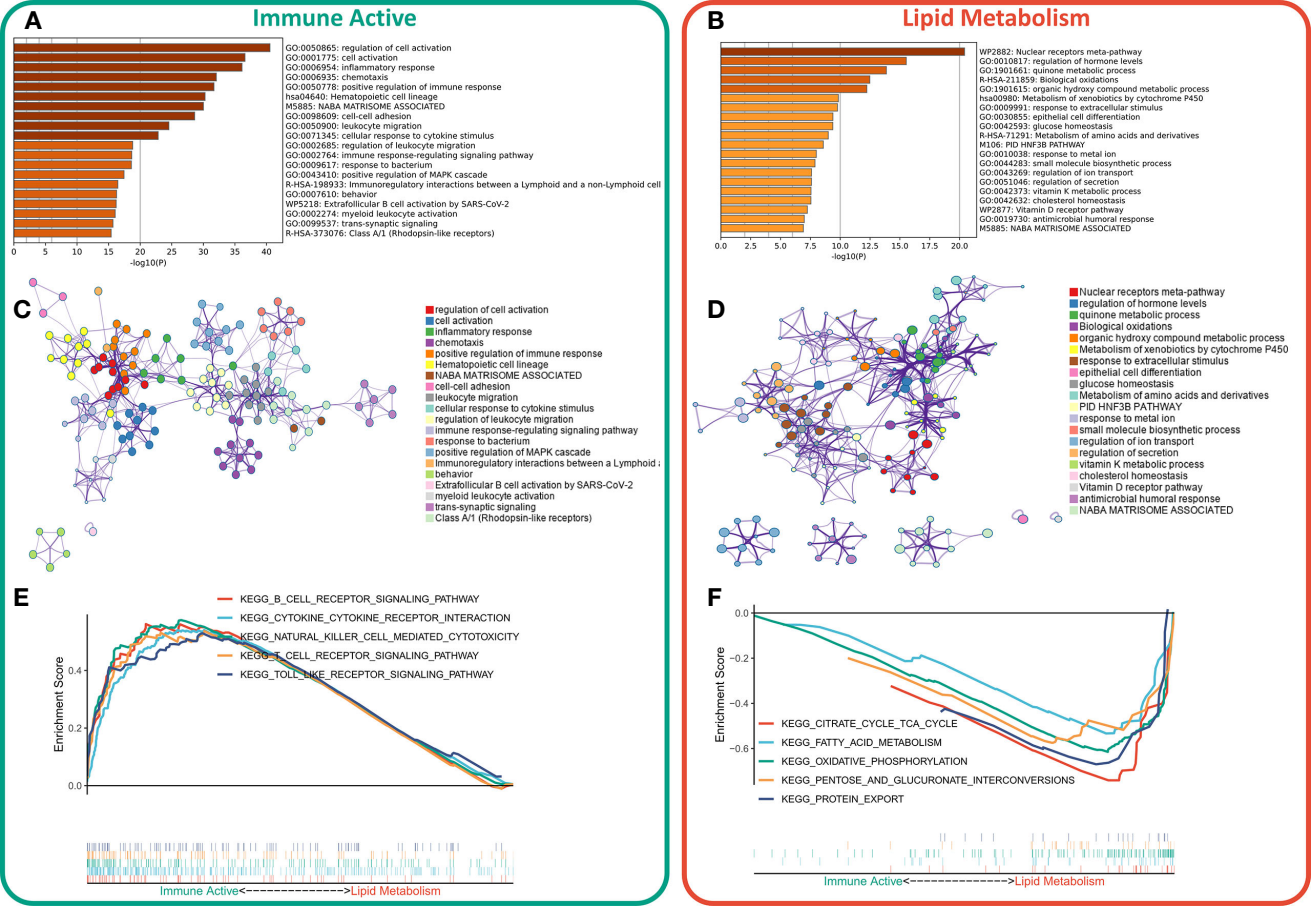
Functional enrichment of different subtypes. **(A)** Functional enrichment of characteristic genes of immune activity subtypes. **(B)** Functional enrichment of lipid metabolism subtype characteristic genes. **(C)** Functional network of immunoactive subtypes. **(D)** Functional network of lipid metabolism subtypes. **(E)** KEGG pathway enriched in immunocompetent subtypes. **(F)** KEGG pathway enriched in lipid metabolism subtypes.

First, the DEGs between the two subtypes were identified by limma package. According to the threshold of FDR < 0.05 and FC > 2, a total of 1597 DEGs were identified, of which 1233 were up-regulated in immunoactive subtypes and 362 were up-regulated in lipid metabolism subtypes. Detailed results are provided in **Table S4**. Based on the functional enrichment analysis, the up-regulated genes in immunoactive subtypes mainly regulate cell activation, inflammatory response, cell adhesion and lymphocyte migration (**Figure 3A**), and **Figure 3C** showed the functional interaction network of immunoactive subtypes. The up-regulated genes in lipid metabolism subtypes mainly regulate biological oxidation, epithelial cell differentiation and glucose homeostasis (**Figure 3B**). **Figure 3D** shows the functional interaction network of lipid metabolism subtypes. GSEA analysis showed that the pathways enriched in immunoactive subtypes were mainly B-cells receptor, T-cells receptor, Toll-like receptor signal pathway and NK-cells killing activity (**Figure 3E**). The pathways enriched in lipid metabolism subtypes were fatty acid metabolism, protein secretion and TCA cycle pathway (**Figure 3F**). In conclusion, these results confirm that the immunocompetent subtype has stronger antitumor immune activity, while the tumor cells of the lipid metabolism subtype have stronger metabolic and proliferative activity, which may lead to the difference in prognosis between the two.

### Difference of immune infiltration between two subtypes

**Figure 4:**
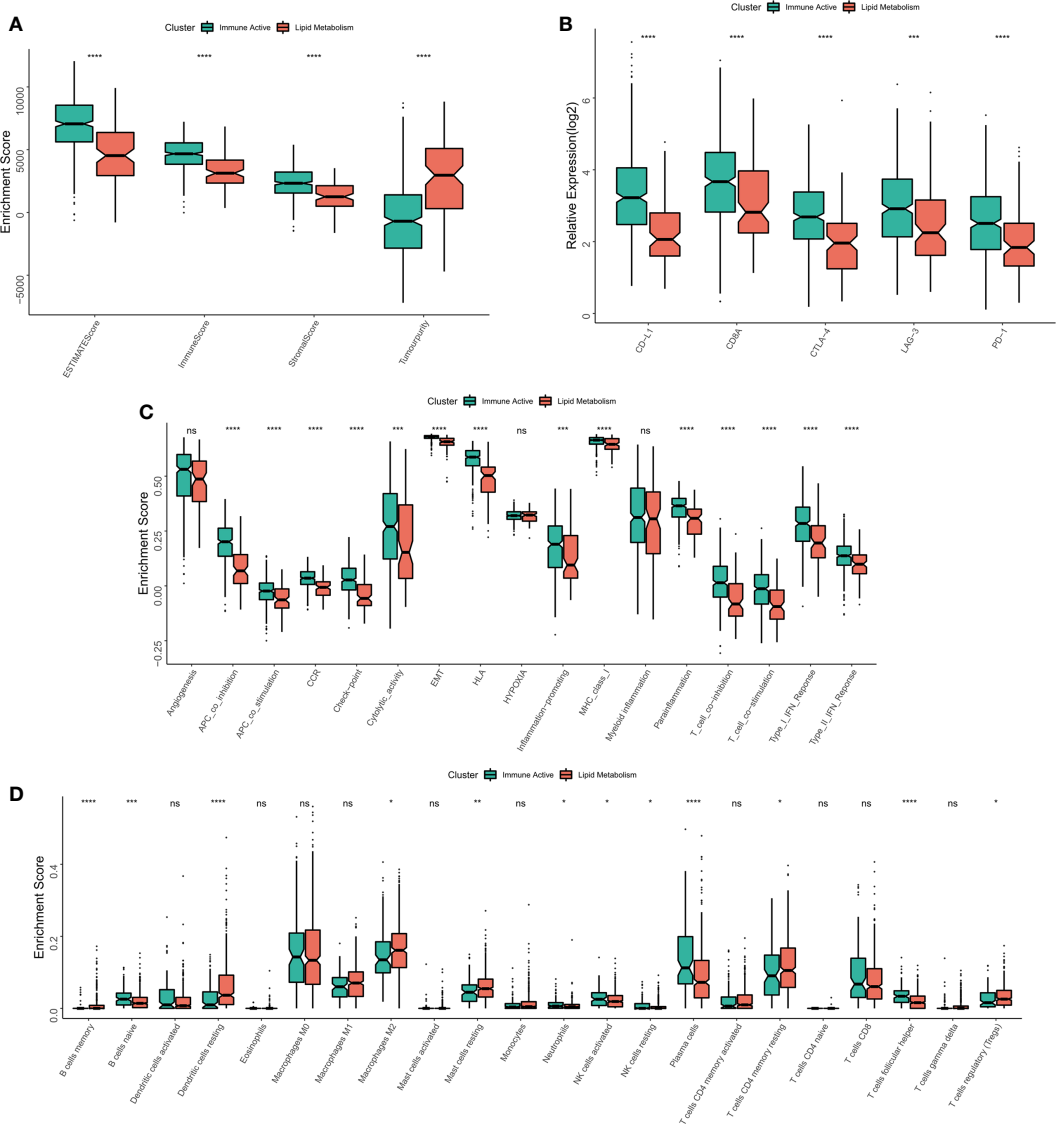
Immune infiltration analysis of different subtypes. **(A)** The difference of Estimate score between the two subtypes. **(B)** Differences in the expression of six typical immune checkpoints (PD-L1, CD8A, CTLA-4, LAG-3, PD-1) between the two subtypes. **(C)** Differences in the activity of immune related pathways between the two subtypes. **(D)** The difference of immune cell infiltration between the two subtypes. *p < 0.05; **p < 0.01; ***p < 0.001; ****p < 0.0001; ns p > 0.05.

The immune infiltration degrees were systematically compared between the two subtypes. First, the estimate algorithm showed that the immune activity subtype had a higher immune score, while the lipid metabolism subtype had a higher tumor purity (**Figure 4A**), which was confirmed in the validation queue (**Figure S3A**). The expression differences between five classical immune checkpoints and therapeutic targets (PD-L1, CD8A, CTLA-4, LAG-3, PD-1) were then examined. The results showed that the five checkpoints were significantly up-regulated in immunoactive subtypes (**Figure 4B**), and the validation cohort (**Figure S3B**). Through ssGSEA algorithm, we found that except for myeloid inflammation, other immune pathways were up-regulated in the immune activity subgroups. In particular, the activity of EMT pathway in the immunoactive pathway was also up-regulated (**Figure 4C**). Similar results were observed in the validation cohort. It is worth noting that the activity of angiogenesis pathway in lipid metabolism subtypes was up-regulated in the validation cohort (**Figure S3C**). Finally, cibersort results showed that NK cells, plasma cells and natural B cells increased in immunoactive subtypes, while Tregs increased in lipid metabolism subtypes (**Figure 4D**). In addition, higher Tregs in lipid metabolism subtypes were also confirmed in the validation cohort (**Figure S3D**). In conclusion, these results convey that the immunoactive subtype has more antitumor immune activity and effector immune cells, while the lipid metabolism subtype is inhibited by higher Tregs infiltration.

### Analysis of genome changes among subtypes

**Figure 5:**
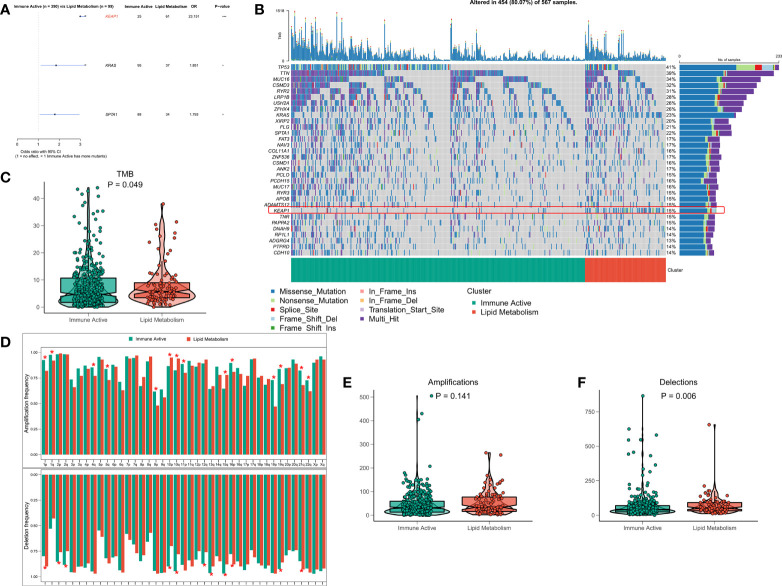
Genome driven events of different subtypes. **(A)** The forest map shows the genes with significant mutation differences between the two subtypes. **(B)** Oncoplot showed the mutation landscape of top25 mutation driver gene among subtypes. **(C)** The difference of TMB between the two subtypes. **(D)** The histogram analyzed the CNV events on the chromosome arm among subtypes. **(E)** Differences in overall amplification events among subtypes. **(F)** Differences in overall missing events among subtypes. *p < 0.05; ***p < 0.001;.

The original mutation data were processed with maftools package. Chi square test showed that the mutation frequency of KEAP1, KRAS and SPTA1 in lipid metabolism subtypes increased, especially KEAP1 (**Figure 5A**). The waterfall diagram shows the mutation map difference of a total of 32 high-frequency mutant genes between the two subtypes (**Figure 5B**). The TMB of each patient was calculated, and the results showed that the lipid metabolism subtype had a higher TMB, but the difference between the two subtypes was not significant (**Figure 5C**). CNV leads to chromosome variation in another way. We then evaluated the correlation between FRS and CNV and found that the amplification and deletion levels of immunoactive subtypes were significantly higher at the chromosome arm level (**Figure 5D**). The box diagram showed no significant difference in the total number of chromosome amplification between the two subtypes (**Figure 5E**), and the number of chromosome deletions in the lipid metabolism subtype increased significantly (**Figure 5F**).

### Immunoactive subtypes that are more sensitive to immunotherapy

**Figure 6:**
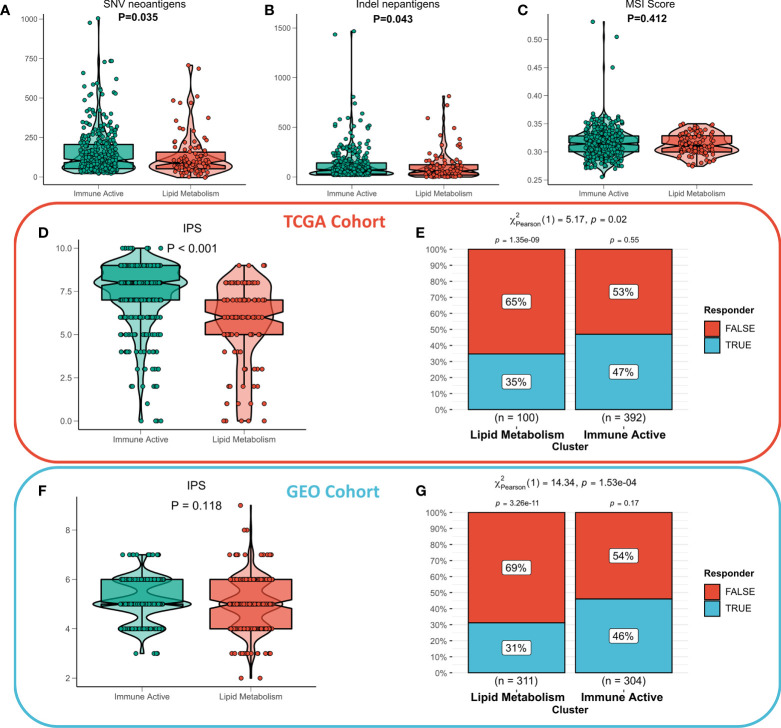
Immunotherapeutic sensitivity of different subtypes. **(A)** Differences in SNV neoantigens between the two subtypes. **(B)** The difference of indel neoantigens between the two subtypes. **(C)** The difference of MSI score between the two subtypes. **(D)** IPS differences between subtypes in TCGA cohort and **(F)** GEO cohort. The TIDE algorithm predicts the immune treatment responses of different subtypes in **(E)** TCGA queue and **(G)** GEO queue.

The functional differences and immune landscape among subgroups suggest that patients with immunoactive subtypes may have better immune treatment response. According to the literature, better immunotherapeutic efficacy is in close relation to the increase in the number of neoantigens (32, 33). Therefore, we first evaluated the difference in the number of neoantigens between the two subtypes, and the results showed that the immunoactive subtypes have more SNV neoantigens and Indel neoantigens (**Figures 6A, B**). Recent studies have shown that MSI score is expected to become a new predictor of immunotherapy (34). However, there is no significant difference in MSI score between the two subtypes (**Figure 6C**). IPS can systematically evaluate the activity of effector immune cells and immune treatment response of patients. The discovery queue showed that IPS of immunoactive subtypes was significantly higher than that of lipid metabolism subtypes (**Figure 6D**), and the response rate of immunoactive subtypes to immunotherapy predicted by TIDE algorithm was higher than that of lipid metabolism subtypes (**Figure 6E**). Although there was no significant difference in IPS between the two subtypes in the validation cohort, the immunoactive subtypes in the validation cohort also had a higher response to immunotherapy (**Figures 6E, F**). In conclusion, our results suggest that immunoactive subtypes are more sensitive to immunotherapy.

### Constructing and validating LIS

**Figure 7:**
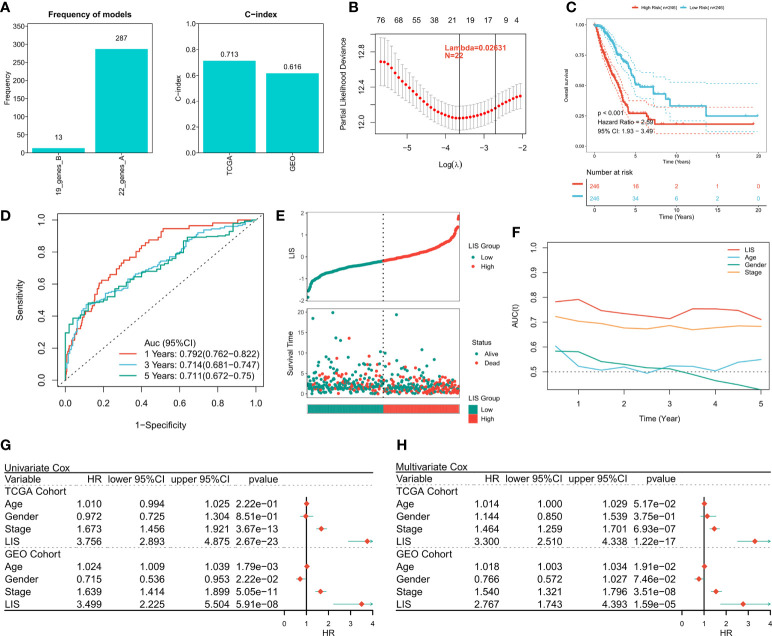
Building and verifying LIS. **(A)** Identifying the best Lasso model. Left: the frequency of different gene combinations in Lasso Cox regression model; Right: the best model is C-index in TCGA and GEO queues. **(B)** According to the best λ = 0.02631, the best 22-genes model was selected. **(C)** Survival curve of high and low LIS subgroups. **(D)** ROC analysis of LIS in TCGA queue. **(E)** The scatter plot shows the survival status of patients with different LIS in the TCGA cohort. **(F)** TROC curve of LIS in TCGA queue. **(G)** Univariate Cox regression analysis of OS in TCGA and GEO cohorts. **(H)** Multivariate Cox regression analysis of OS in TCGA and GEO cohorts.

First, 1597 DEGs were analyzed by univariate Cox regression to identify the prognostic valuable DEGs. According to the threshold of p < 0.001, a total of 88 DEGs with prognostic significance were identified. Then, these 88 DEGs were recruited for Lasso regression to simplify the model. After 300 iterations, the model with 22 DEGs was the most stable showing a suitable efficacy in the training queue as well as the validation queue (C index > 0.6, **Figure 7A**). According to the best λ (0.02631), the best model of 22 genes was identified (**Figure 7B**), and LIS was generated according to the formula: *LIS* = ∑*iCoefficient*(*mRNA_i_*) × *Expression*(*mRNA_i_*), detailed coefficients of 22 LIS genes can be found in **Table S5**. According to the survival analysis, patients with high LIS showed significantly less survival rate compared to the patients with low LIS (p < 0.001, **Figure 7C**), which was confirmed in the validation cohort (p < 0.001, **Figure S4A**). Based on the ROC analysis, the AUC values of the model in 1 year, 3 years, and 5 years were 0.792, 0.714, and 0.711, respectively (**Figure 7D**). In the external validation queue, LIS also had satisfactory prediction efficiency, specifically, 0.68 in 1 year, 0.69 in 3 years, 0.69 in 5 years and 0.71 in 8 years (**Figure S4B**). **Figure 7E** shows that the survival status of patients with high LIS were significantly worse compared that of patients with low LIS, and similar results were observed in the validation cohort (**Figure S4C**). TROC analysis showed that LIS was the best predictor of OS (**Figure 7F**), and the effectiveness of LIS and Stage was equivalent in the validation queue (**Figure S4D**). Finally, univariate Cox regression confirmed that LIS was an independent prognostic indicator in both training and validation sets (p < 0.0001, **Figure 7G**). Multivariate Cox regression showed that LIS was still an independent prognosticator for OS in the training and validation cohorts after correcting for other factors (p < 0.0001, **Figure 7H**).

### Quantifying the risk of individual LUAD patients

**Figure 8:**
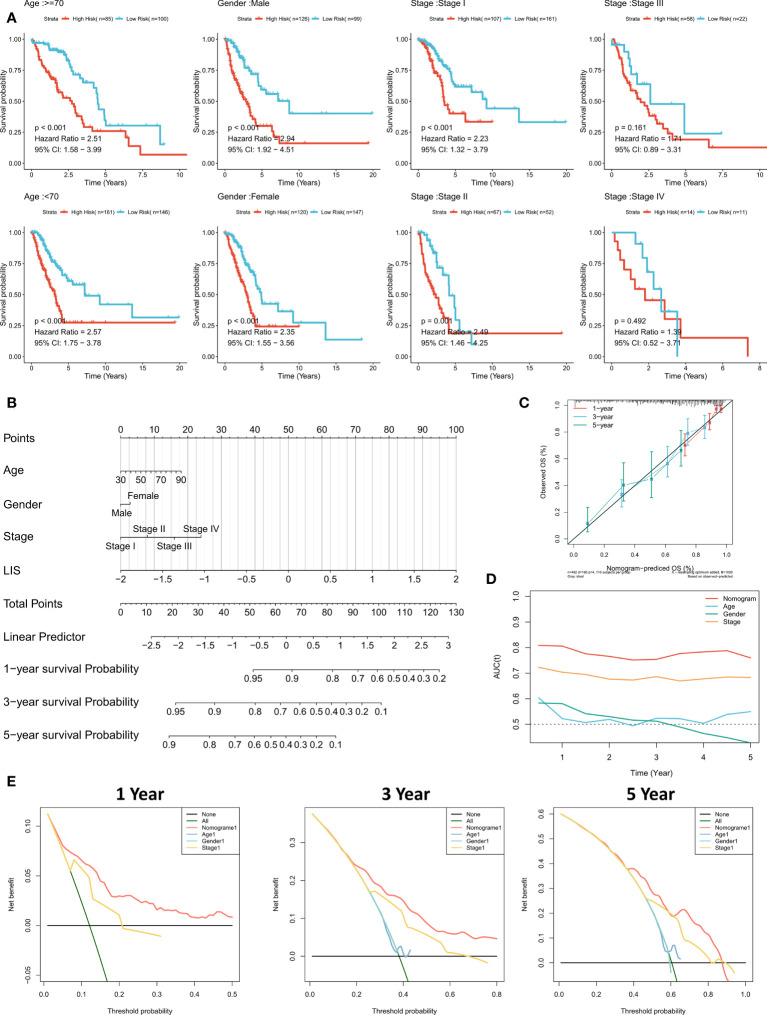
Quantifying individual LIS related risks. **(A)** Subgroup analysis of LIS in patients with different clinical characteristics. **(B)** LIS based nomograms were used to quantify individual patient risks. **(C)** Nomogram calibration curves at 1, 3 and 5 years. **(D)** TROC curve of nomogram. **(E)** Nomogram DCA curve in 1, 3 and 5 years.

Subgroup analysis showed that LIS in the training cohort showed excellent predictive ability in different clinical subgroups except patients in stage 3 and stage 4 (p < 0.001, **Figure 8A**). In the validation cohort, LIS was able to distinguish patients with poor survival except for patients in stage 2-4 (p < 0.05, **Figure S4E**). These results suggest that LIS shows better performance in predicting early LUAD patients. For better quantifying of the death risk in individual LUAD patients, nomograms were constructed based on LIS (**Figure 8B**). Nomogram correction curve shows that nomogram model has good stability and accuracy in 1, 3 and 5 years (**Figure 8C**). TROC analysis showed that compared with clinical characteristics, nomogram model was the best predictor (**Figure 8D**). DCA was then performed to calculate the decision-making benefits of nomogram model. The results showed that nomogram was suitable for risk assessment of LUAD patients in 1, 3 and 5 years (**Figure 8E**).

### LIS in predicting immunotherapy

**Figure 9:**
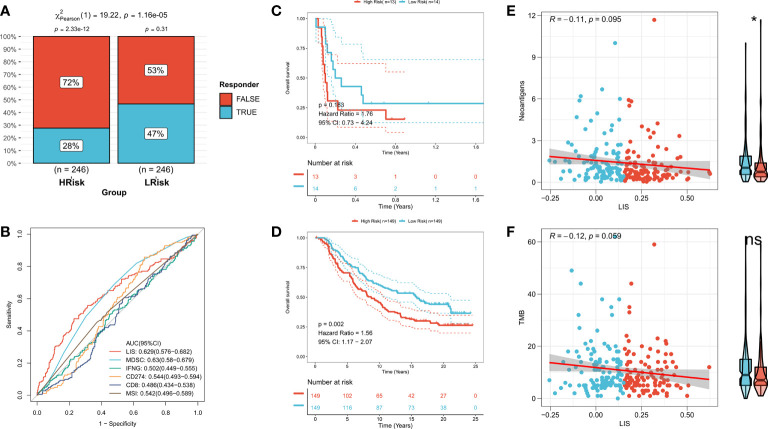
LIS in predicting immunotherapy. **(A)** TIDE algorithm predicted the response rate of immunotherapy with high LIS and low LIS. **(B)** ROC curve shows the prediction accuracy of LIS and different immune markers. **(C)** Survival curve of high LIS and low LIS subgroups in GSE135222 cohort. **(D)** Survival curve of high LIS and low LIS subgroups in Imvigor210 cohort. **(E)** Correlation between LIS and neoantigens in Imvigor210 queue. **(F)** Correlation between LIS and TMB in Imvigor210 queue. *p < 0.05; ns p > 0.05.

First, TIDE was used to evaluate the difference of immunotherapy response between patients with high LIS and patients with low LIS. According to the results, patients with low LIS showed to be more benefitting from immunotherapy (**Figure 9A**, **Figure S5A**). Then five widely used immunotherapy biomarkers were calculated, including MDSC, MSI score, IFNG, CD8 and CD274. In the training cohort and validation cohort, LIS provided higher accuracy in predicting immunotherapy (**Figure 9B**, **Figure S5B**). Then, two immunotherapy cohorts were included to further study whether LIS could predict patients’ response to immunotherapy. Consistent with the above, patients with high LIS showed worse survival in these two immunotherapy cohorts (**Figures 9C, D**). Finally, the relationship between LIS and neoantigens and TMB in Imvigor210 cohort was evaluated. The results showed that LIS had no strong correlation with neoantigens and TMB. However, patients with low LIS had higher neoantigens (**Figures 9E, F**). Overall, our study strongly confirms LIS as a prognosis factor for OS and immunotherapeutic response of patients, and is superior to widely used biomarkers.

## Discussion

Lung cancer is the main cause of cancer-related death and LUAD is the most common histological subtype with the most patients at the advanced stage on the initial diagnosis (35, 36). Although a variety of targeted therapies and new chemotherapeutic drugs have been approved, the OS of advanced patients is still not ideal (4). Lipid metabolism has long been reported as the main energy source of cancer cells and is involved in the incidence and development of cancer (8). Recently, the dual regulation of lipid metabolism on immune response in TME has attracted extensive attention and has become a promising target for targeted therapy (6, 10). Our study identified and verified two heterogeneous subtypes in LUAD, one of which was an effector immune cell with more expression of IRGs, enrichment of immunoactive pathways and high abundance, which was defined as an immunoactive subtype. Another kind of suppressive immune cells expressing more LMGs and high abundance, showing the characteristics of tumor metabolism and proliferation, was defined as lipid metabolism subtype. We verified the stability and repeatability of the two subtypes in a GEO meta cohort. These two subtypes also showed heterogeneity in genome driven events, clinical outcomes, and immunotherapy responses. In addition, a robust prognostic feature was proposed based on these two subtypes: LIS. Further analysis showed that LIS shows a leading advantage in predicting the immunotherapy of LUAD patients. These results promote the understanding of the interaction between lipid metabolism and TME and offer a new direction for clinical management and precision treatment of LUAD patients. These two subtypes showed different clinical characteristics. The survival of immunoactive phenotype was significantly better than that of lipid metabolism phenotype, and patients with more disease progression were in lipid metabolism phenotype. Functional enrichment indicated that metabolic related pathways and cell-cycle related pathways were enriched in the lipid metabolism phenotype, while effector immune cell receptor signaling pathways and immune related pathways were enriched in the immunoactive phenotype. In addition, immune infiltration analysis also suggested that there was higher effector immune cell infiltration in the immunoactive phenotype, and more tumor cells and inhibitory immune cells in the lipid metabolism subtype. These results suggest the hypermetabolism and proliferation of tumors in the lipid metabolism subtype and explain the worse survival rate and tumor progression of patients with this subtype (37). More effector immune cells and stronger immune activity in the immunoactive phenotype play an anti-tumor role, resulting in better survival and tumor remission of patients (38).

Next, in order to elaborate the molecular characteristics of the two subtypes, the genomic alterations of the two subtypes were compared. In general, there was a higher TMB in the lipid metabolism subtype. It is worth noting that the mutation frequency of KEAP1 gene in the lipid subtype was significantly increased compared to that in the immunoactive phenotype. KEAP1 is an essential regulator of cell homeostasis and antioxidant stimulation (39). Studies have reported that this mutation is common in NSCLC with close correlation to higher tumor growth and invasiveness (40). Additionally, tumors bearing KEAP pathway mutations have been reported in preclinical and clinical studies, which have stronger resistance to traditional treatment methods, such as chemotherapy, radiotherapy, and targeted therapy (41–43). In addition, we found that the amplification and deletion levels of immunoactive subtypes were significantly higher at the chromosome arm level, and the deletion levels of lipid metabolism subtypes were higher in general. The contradictory results may suggest that CNV does not seem to be playing a pivotal role in regulating the differences between subtypes. In general, the genomic changes of these two subtypes are mainly mediated by gene mutations, especially KEAP1, which may contribute to the heterogeneous response of subtypes to tumor treatment, leading to different clinical outcomes. In addition, KEAP1 may also be a new target for drug development and clinical treatment of LUAD.

Finally, a prognostic feature called LIS was developed and validated in the TCGA cohort, GEO meta cohort, and two external immunotherapy cohorts. High LIS is an independent negative prognostic factor for OS, and subgroup analysis showed that LIS showed stronger performance in predicting early LUAD patients. Considering the heterogeneity of subtypes in immunotherapy, we also evaluated the effectiveness of LIS in predicting immunotherapy. The results showed that LIS also showed high accuracy in the immunotherapy cohort. In addition, LIS also showed better accuracy than commonly used biomarkers (MDSC, MSI score, IFNG, CD8 and CD274). Finally, we found that patients with low LIS may have more neoantigens, which may lead to stronger immunotherapy sensitivity in patients with low LIS. In conclusion, our results suggest that LIS is not only a robust prognostic marker, but also a promising predictive marker of immunotherapy.

We admit that our research also has some defects. First, we only use Bulk-seq data without considering the heterogeneity between cells. Secondly, the sequenced samples came from tumor tissue, which may lead to the fact that LIS is not suitable for peripheral blood samples, and the clinical application is limited. Finally, although we used algorithms and mature immunotherapy cohorts to evaluate the sensitivity of the two subtypes to immunotherapy, prospective clinical research cohorts are still needed for validation. In conclusion, our work identified and validated heterogeneous lipid metabolism subtypes and immune activity subtypes in LUAD, which showed heterogeneity in clinical outcomes, biological functions, immune infiltration, and genome driven events. In addition, we have developed a feature called LIS, which can be used as a reliable prognostic biomarker for predicting OS and immunotherapy response. These results promote the understanding of the interaction between lipid metabolism and TME and offer a new direction for clinical management and precision therapy of LUAD patients.

## Data availability statement

The original contributions presented in the study are included in the article/**Supplementary Material**. Further inquiries can be directed to the corresponding authors.

## Author contributions

XG performed the data analyses and wrote the manuscript. SW contributed significantly to analysis and manuscript preparation. ZL helped perform the analysis with constructive discussions. HX contributed to the conception of the study. All authors contributed to the article and approved the submitted version.

## Funding

This work was supported by the Programs of Shanghai Pulmonary Hospital (No. fkxr1902 and No.fkxr1904).

## Acknowledgments

We greatly appreciate the analytical data provided by the TCGA and GEO databases.

## Conflict of interest

The authors declare that the research was conducted in the absence of any commercial or financial relationships that could be construed as a potential conflict of interest.

## Publisher’s note

All claims expressed in this article are solely those of the authors and do not necessarily represent those of their affiliated organizations, or those of the publisher, the editors and the reviewers. Any product that may be evaluated in this article, or claim that may be made by its manufacturer, is not guaranteed or endorsed by the publisher.

## Supplementary material

Supplementary Figure 1 The workflow of this study. In this study, we aimed to survey the crosstalk between lipid metabolism and tumor immune response in LUAD patients and identified two heterogeneous subtypes (lipid metabolism subtype and immune activity subtype). These two subtypes show specific differences in clinical outcomes, biological functions, immune infiltration and genomic variation. In addition, a lipid-immune score (LIS) was developed and validated, which shows significant advantages in predicting prognosis and immunotherapy response. In conclusion, our work strengthens the understanding of the complex role between lipid metabolism and immune system in LUAD and provides a new perspective and reference for the accurate prediction and immunotherapy of LUAD patients.

Supplementary Figure 2 Validation of immune and lipid subtypes. **(A)** CDF curve of consensus matrix of different K in GEO queue. **(B)** Consensus matrix when k = 2 in GEO queue. **(C)** Expression heat maps of LMGs and IRGs in two subtypes in the GEO cohort. **(D)** Survival curve of two subtypes in GEO cohort.

Supplementary Figure 3 Verification of immune infiltration in subtypes. **(A)** Differences in Estimate scores between the two subtypes in the GEO cohort. **(B)** Differences in the expression of six typical immune checkpoints (PD-L1, CD8A, CTLA-4, LAG-3, PD-1) between the two subtypes in the GEO cohort. **(C)** Differences in immune related pathway activity between the two subtypes in the GEO cohort. **(D)** Differences in immune cell infiltration between the two subtypes in the GEO cohort.

Supplementary Figure 4 External verification of LIS. **(A)** Survival curves of high and low LIS subgroups in the GEO cohort. **(B)** ROC analysis of LIS in GEO queue. **(C)** The scatter plot shows the survival status of different LIS patients in the GEO cohort. **(D)** TROC curve of LIS in GEO queue. **(E)** Subgroup analysis of LIS in patients with different clinical characteristics in GEO cohort.

Supplementary Figure 5 External verification of LIS. **(A)** The TIDE algorithm in GEO queue predicted the response rate of immunotherapy with high LIS and low LIS. **(B)** ROC curves in the GEO cohort showed the predictive accuracy of LIS and different immune markers.
