# Supplementary figures and images for: Machine learning reveals two heterogeneous subtypes to assist immune therapy based on lipid metabolism in lung adenocarcinoma
Source: Front Immunol. 2022 Sep 27;13:1022149. doi: 10.3389/fimmu.2022.1022149 (PMC9551187; doi:10.3389/fimmu.2022.1022149)

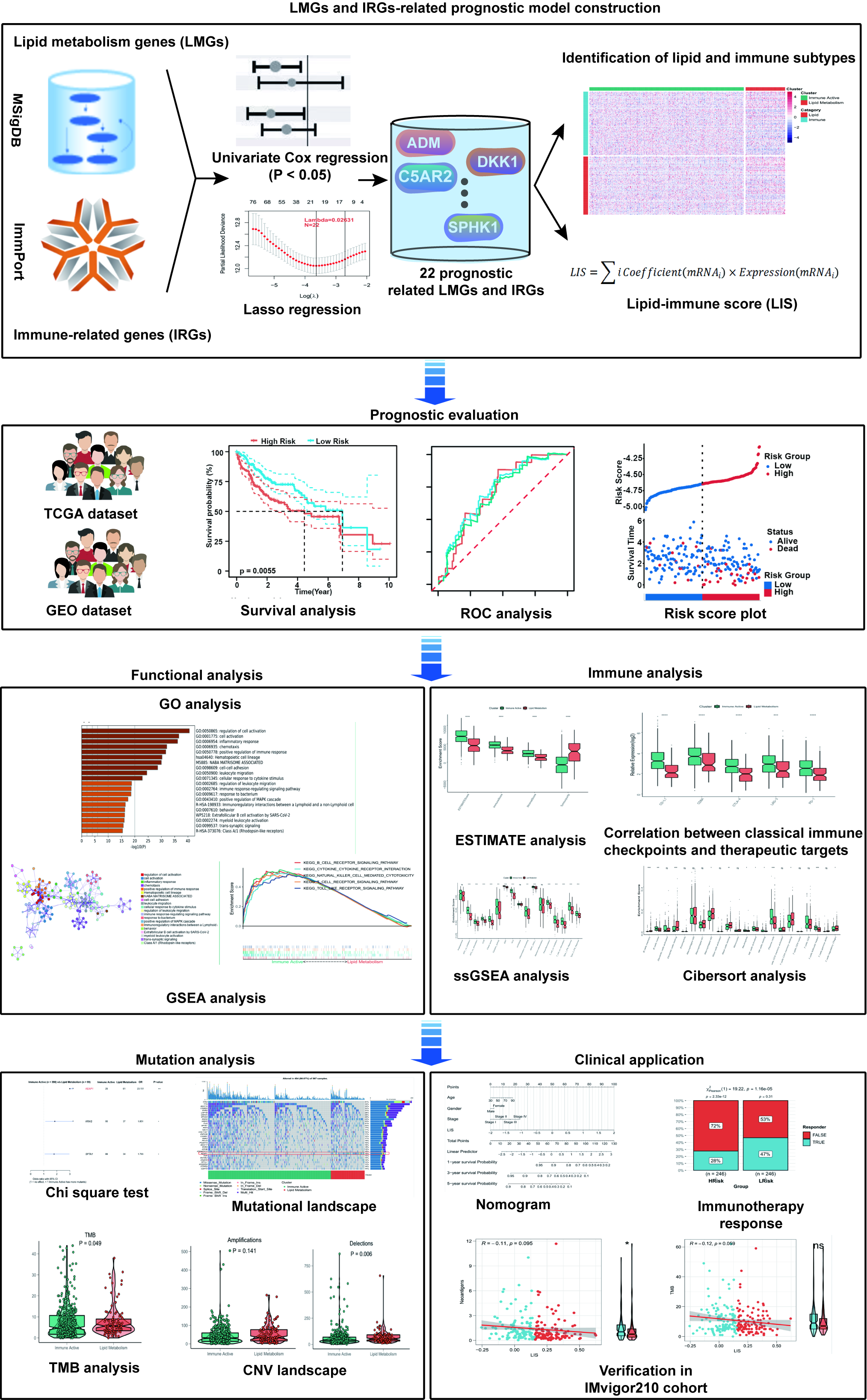

Supplement: Supplementary Figure 1 — The workflow of this study. In this study, we aimed to survey the crosstalk between lipid metabolism and tumor immune response in LUAD patients and identified two heterogeneous subtypes (lipid metabolism subtype and immune activity subtype). These two subtypes show specific differences in clinical outcomes, biological functions, immune infiltration and genomic variation. In addition, a lipid-immune score (LIS) was developed and validated, which shows significant advantages in predicting prognosis and immunotherapy response. In conclusion, our work strengthens the understanding of the complex role between lipid metabolism and immune system in LUAD and provides a new perspective and reference for the accurate prediction and immunotherapy of LUAD patients. [file Image_1.tif]

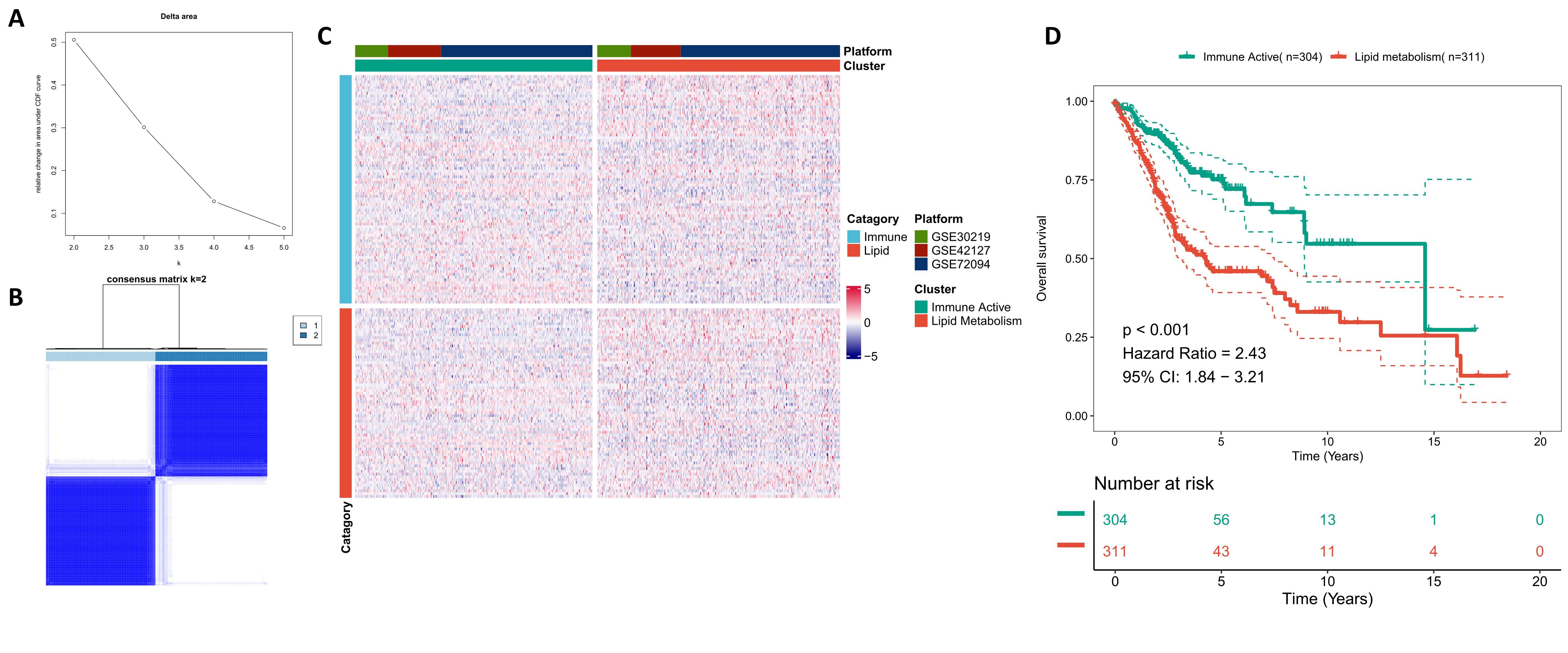

Supplement: Supplementary Figure 2 — Validation of immune and lipid subtypes. (A) CDF curve of consensus matrix of different K in GEO queue. (B) Consensus matrix when k = 2 in GEO queue. (C) Expression heat maps of LMGs and IRGs in two subtypes in the GEO cohort. (D) Survival curve of two subtypes in GEO cohort. [file Image_2.tif]

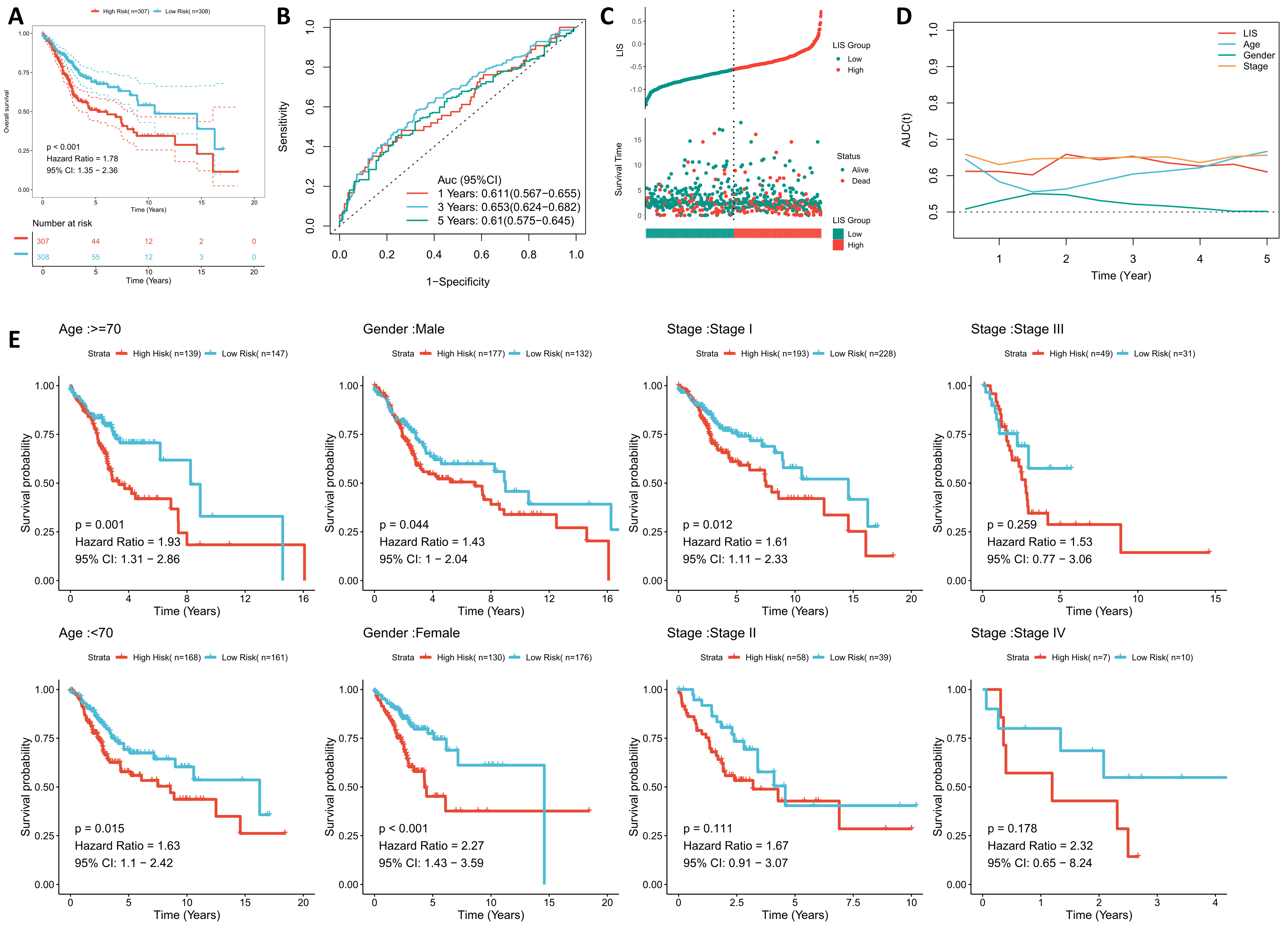

Supplement: Supplementary Figure 3 — Verification of immune infiltration in subtypes. (A) Differences in Estimate scores between the two subtypes in the GEO cohort. (B) Differences in the expression of six typical immune checkpoints (PD-L1, CD8A, CTLA-4, LAG-3, PD-1) between the two subtypes in the GEO cohort. (C) Differences in immune related pathway activity between the two subtypes in the GEO cohort. (D) Differences in immune cell infiltration between the two subtypes in the GEO cohort. [file Image_3.tif]

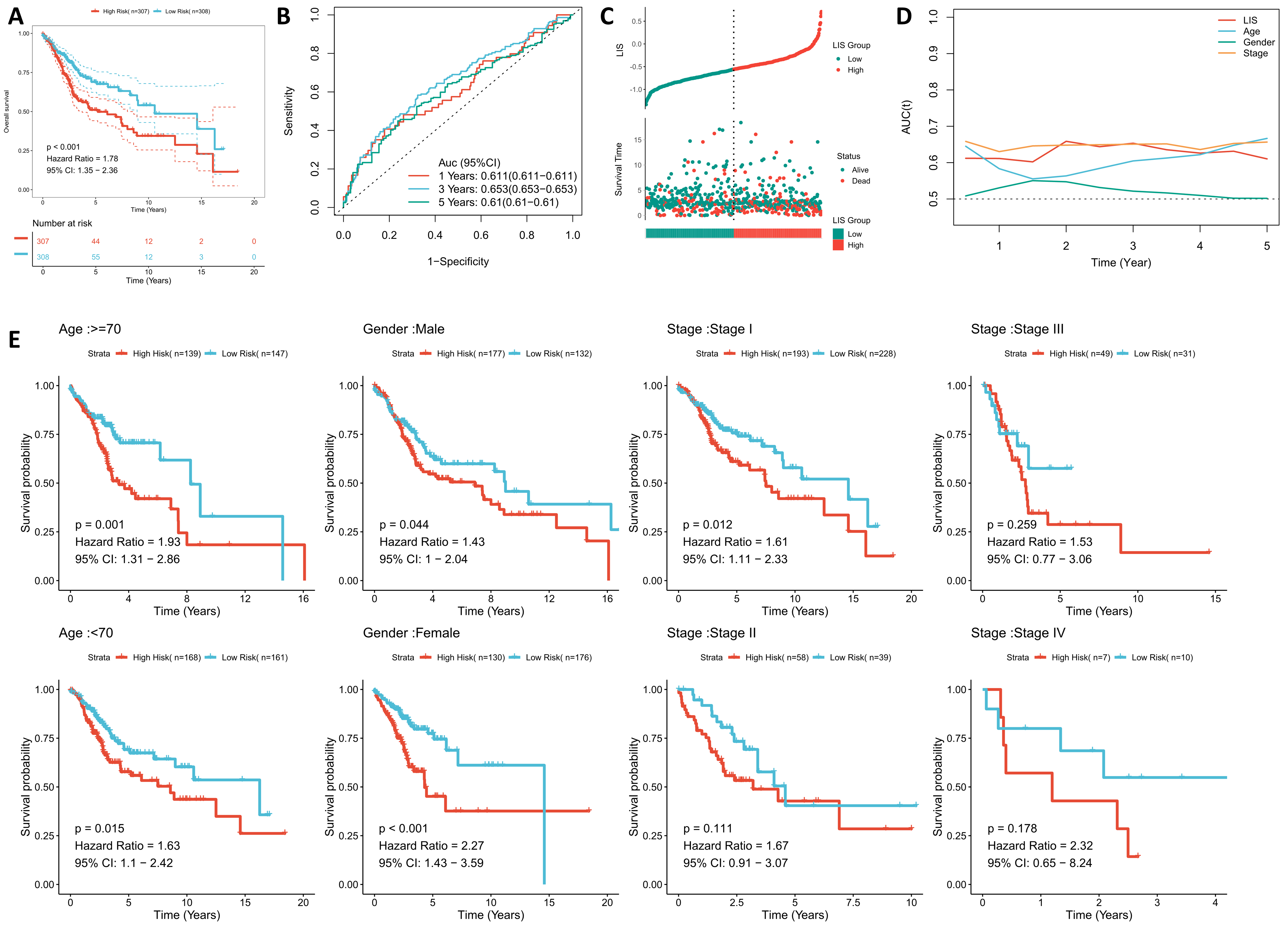

Supplement: Supplementary Figure 4 — External verification of LIS. (A) Survival curves of high and low LIS subgroups in the GEO cohort. (B) ROC analysis of LIS in GEO queue. (C) The scatter plot shows the survival status of different LIS patients in the GEO cohort. (D) TROC curve of LIS in GEO queue. (E) Subgroup analysis of LIS in patients with different clinical characteristics in GEO cohort. [file Image_4.tif]

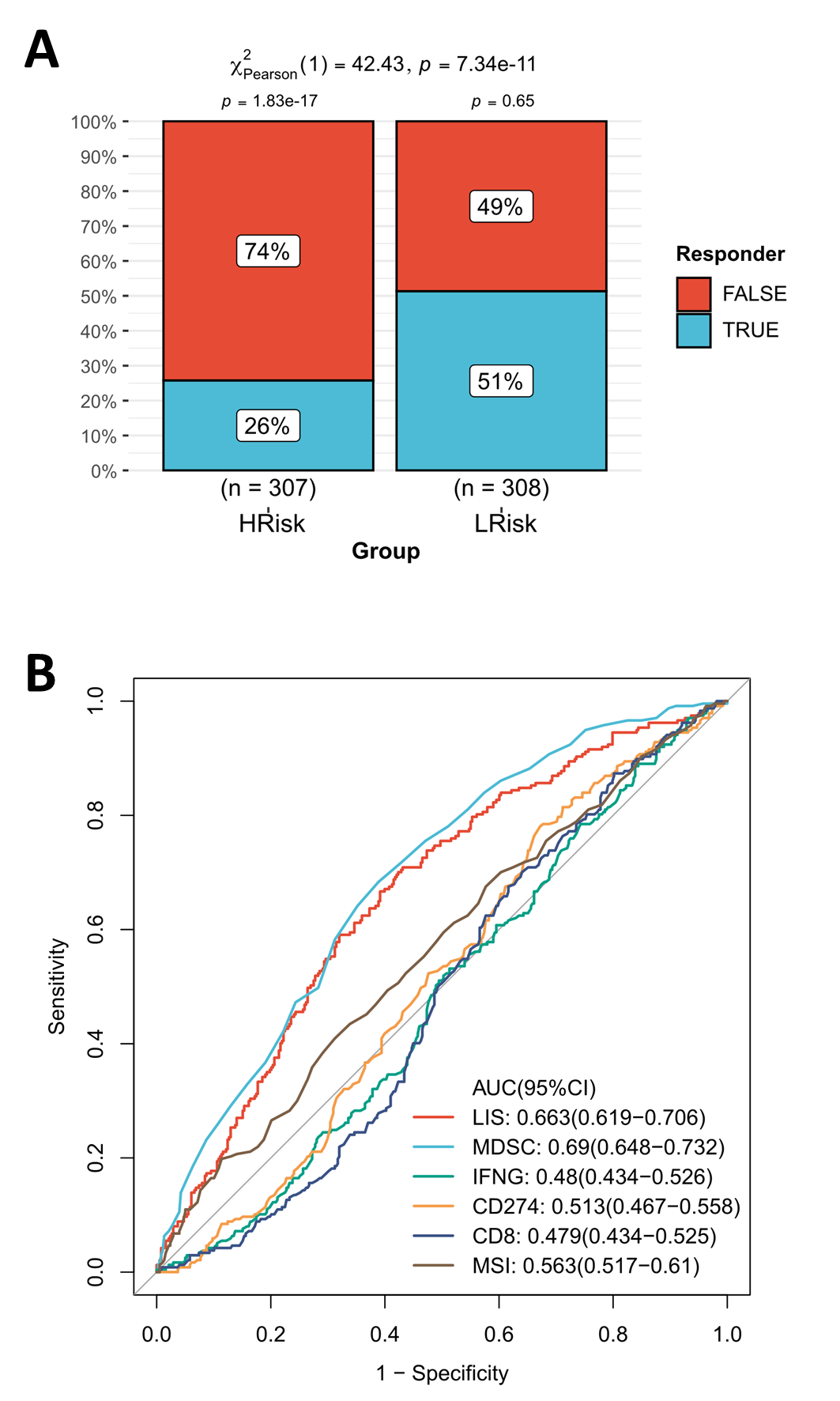

Supplement: Supplementary Figure 5 — External verification of LIS. (A) The TIDE algorithm in GEO queue predicted the response rate of immunotherapy with high LIS and low LIS. (B) ROC curves in the GEO cohort showed the predictive accuracy of LIS and different immune markers. [file Image_5.tif]
